# Immunometabolism Modulation by Extracts from Pistachio Stalks Formulated in Phospholipid Vesicles

**DOI:** 10.3390/pharmaceutics15051540

**Published:** 2023-05-19

**Authors:** Simone Pani, Ilaria Pappalardo, Anna Santarsiero, Antonio Vassallo, Rosa Paola Radice, Giuseppe Martelli, Francesco Siano, Simona Todisco, Paolo Convertini, Carla Caddeo, Vittoria Infantino

**Affiliations:** 1 Department of Scienze della Vita e dell’Ambiente, University of Cagliari, S.P. Monserrato-Sestu Km 0.700, 09042 Monserrato, Italy; simone.pani@unica.it; 2 Department of Science, University of Basilicata, Viale dell’Ateneo Lucano 10, 85100 Potenza, Italy; ilaria.pappalardo8@gmail.com (I.P.); santarsieroanna90@gmail.com (A.S.); antonio.vassallo@unibas.it (A.V.); rosapaolaradice@gmail.com (R.P.R.); giuseppe.martelli@unibas.it (G.M.); simona.todisco@unibas.it (S.T.); paolo.convertini@gmail.com (P.C.); vittoria.infantino@unibas.it (V.I.); 3 Spinoff TNcKILLERS s.r.l., Viale dell’Ateneo Lucano 10, 85100 Potenza, Italy; 4 Institute of Food Science, National Research Council, Via Roma 64, 83100 Avellino, Italy; francesco.siano@isa.cnr.it

**Keywords:** pistachio stalks, essential oil, hydrolate, phospholipid vesicles, skin delivery, activated macrophages, immunometabolism, inflammatory mediators, citrate pathway

## Abstract

Several studies have demonstrated the effectiveness of plant extracts against various diseases, especially skin disorders; namely, they exhibit overall protective effects. The Pistachio (*Pistacia vera* L.) is known for having bioactive compounds that can effectively contribute to a person’s healthy status. However, these benefits may be limited by the toxicity and low bioavailability often inherent in bioactive compounds. To overcome these problems, delivery systems, such as phospholipid vesicles, can be employed. In this study, an essential oil and a hydrolate were produced from *P. vera* stalks, which are usually discarded as waste. The extracts were characterized by liquid and gas chromatography coupled with mass spectrometry and formulated in phospholipid vesicles intended for skin application. Liposomes and transfersomes showed small size (<100 nm), negative charge (approximately −15 mV), and a longer storage stability for the latter. The entrapment efficiency was determined via the quantification of the major compounds identified in the extracts and was >80%. The immune-modulating activity of the extracts was assayed in macrophage cell cultures. Most interestingly, the formulation in transfersomes abolished the cytotoxicity of the essential oil while increasing its ability to inhibit inflammatory mediators via the immunometabolic citrate pathway.

## 1. Introduction

Pistachios (*Pistacia vera* L.) are mainly produced in the United States (USA), Iran, and Turkey. In Italy, which is among the top seven pistachio producers in the world, pistachios are cultivated in the southern regions. Pistachios are a source of fibers, minerals, vitamins, fats (50–60%), and unsaturated fatty acids. They are also rich in phenolic compounds and have recently been ranked among the top 50 food products with the highest antioxidant potential [1,2]. Polyphenols, including flavonoids, are widely present in all parts of pistachio [3]. Emerging evidence suggests that pistachio flavonoids play a beneficial role in cognitive performance, as they can lower the risk of memory and cognitive decline in the long- term [4]. The daily intake of flavonoids has been associated with lower probability of cognitive decline. Supplementing a regular diet with pistachios was found to reduce lipid dysmetabolism, oxidative stress, and mitochondrial dysfunction [4]. In another study, 15 lipophilic extracts from *P. vera* were screened for their antimicrobial and antiviral activities against various strains. Remarkable antifungal activity against *Candida albincas* and *Candida parapsilosis*, greater antibacterial activity against gram-positive bacteria, and antiviral activity against herpes simplex virus and parainfluenza viruses were demonstrated [5].

Several in vitro and in vivo studies have probed the antioxidant effects of pistachio by comparing the activity of different parts of the nut or of different types of extract. Polyphenols from pistachios were found to be able to exert anti-inflammatory activity and protect against oxidative stress, reducing the expression of the markers of nitrosative stress (e.g., iNOS and COX2) and reducing NO formation; this suggests a potential therapeutic use of pistachio products [6]. Another in vitro study showed the potential healing effect of extracts from the shells, resins, and galls of *P. vera* [7]. The active compound, 3-epimasticadienolic acid, isolated from *P. vera* shells, was found to significantly increase NIH/3T3 murine fibroblast proliferation and migration in a scratch test, resulting in a reduction of approximately 45% in the scratch area. 3-Epimasticadienolic acid also exhibited a strong inhibitory effect on IL-6 and TNF-α gene expression and a stimulating effect on NF-κB gene expression, resulting in anti-inflammatory action [7].

It has recently been suggested that the regulation of immunometabolic targets could be an effective approach to counteracting infections and inflammatory conditions [8]. The metabolic reprogramming of activated (M1) macrophages requires the activation of the cytosolic ATP citrate lyase (ACLY), which cleaves the citrate into acetyl-CoA and oxaloacetate (OAA). Acetyl-CoA can be used for fatty acid synthesis, arachidonic acid production, and protein acetylation [9]. Moreover, the ACLY nuclear translocation observed in M1 macrophages drives a gene expression reprogramming via NF-κB and histone acetylation [10]. OAA is first transformed into malate through the cytosolic malate dehydrogenase 1. Subsequently, malic enzyme 1 converts malate to pyruvate with the production of nicotinamide adenine dinucleotide phosphate (NADPH). Notably, both iNOS and NADPH oxidase require ACLY-dependent NADPH to synthesize NO• and ROS inflammatory mediators, respectively [11,12]. Therefore, ACLY is upregulated in M1 macrophages and in pathological contexts [13,14].

Hence, considering the anti-inflammatory properties of pistachio derivatives, we theorized a potential use for modulating the activities of macrophages activated in injured skin. However, it is known that, despite their numerous pharmacological properties, natural compounds often suffer from toxicity, poor solubility, and low bioavailability, which considerably reduce their application in therapy. Consequently, researchers have been striving to develop delivery systems capable of solving these issues. Phospholipid vesicles comprise a suitable system. They are versatile, biocompatible nanocarriers that are capable of loading compounds with varied physicochemical properties, protecting them from degradation, increasing their solubility, modulating their release, and facilitating their transport through biological membranes [15,16].

In this study, two pistachio extracts were produced from stalks, which are usually discarded as waste. The extracts were characterized by liquid and gas chromatography–mass spectrometry and formulated in phospholipid vesicles tailored for skin application. The vesicles, namely, liposomes and transfersomes, were characterized for size, surface charge, and storage stability. Their entrapment efficiency was evaluated via the quantification of the major compounds. Furthermore, their immunometabolic-mediated activity was assayed in cultured macrophages.

## 2. Materials and Methods

### 2.1. Extract Preparation

The extracts were obtained from pistachios (*P. vera* L.) harvested in Basilicata (Italy) in September 2020. Different parts of the plant were collected for taxonomic study and specific classification. These materials were deposited at Herbarium Lucanum and were recorded with the following ID number: HLUC 13887. They are available for future study. The pistachio stalks were separated from the rest of the fruit and brought to the laboratory for processing. A total of 2.5 kg of fresh stalks were steam-distilled for 90 min using a Clevenger-type apparatus (with 2 L of water). The steam, which came from a high-pressure boiler, was conveyed upward to the sample. The passage of vapor through the sample favored the extraction of volatile compounds, which were transported to a glass condenser. The essential oil and the hydrolate were separated.

From 2.5 kg of stalks, 7.5 mL of essential oil and 1 L of hydrolate were obtained.

### 2.2. LC-ESI/Quadrupole-Orbitrap/MS Analysis

An HPLC system (Ultimate 3000, Thermo Fisher Scientific, Milan, Italy) coupled to an electrospray ionization source with a high-resolution mass spectrometer (Q Exactive Quadrupole-Orbitrap, Thermo Fisher Scientific) was used to identify the metabolites in *P. vera* extracts. Separation was achieved using a Luna C18 column (Phenomenex, 150 × 2.0 mm, 3 µm), eluting with a mixture of eluent A (ultrapure water-0.1% *v*/*v* formic acid) and eluent B (ultrapure acetonitrile-0.1% *v*/*v* formic acid) using the following gradient: from 5 to 95% of B in 30 min. The injection volume was 10 µL at a flow rate of 0.2 mL/min. MS settings were the following: in positive ion mode, source voltage: 3 kV; capillary temperature: 320 °C; and flow rate of the sheath gas and auxiliary gas: 35.0 and 15.0 arbitrary units. MS spectra were acquired by full-range acquisition covering the 100–300 *m*/*z* range. Data were acquired and processed by Xcalibur 2.2 SP1 software (Thermo Fisher Scientific). For fragmentation studies, a data-dependent scanning was performed by selecting precursor ions as the most intense peaks in LC-MS chromatograms. The identification of metabolites was based on retention times, mass measurements, MS/MS data, exploration of spectral libraries and public repositories for MS-based metabolomic analysis [17], and comparison with data reported in the literature [18,19,20,21,22,23].

### 2.3. GC-FID and GC-MS Characterization of P. vera Essential Oil

A GC system (Agilent 7890, Palo Alto, CA, USA) equipped with a flame ionization detector (FID) with split/splitless capillary inlet system was used. The autosampler (G4513A, Agilent) had a 10 µL syringe set at 1 µL delivery volume at fast injection speed (split ratio 1:30). Analyses of the essential oil (diluted 1:100 with ethanol) were carried out on a 30 m × 0.25 mm id fused silica capillary column with 0.25 µm film of 5% diphenyl:95% dimethylpolixyloxane (Restek Co., Bellefonte, PA, USA). Data acquisition was performed with a ChemStation software v. B04.03 (Agilent). Carrier gas was helium at a flow rate of 1.0 mL/min; make-up gas was nitrogen at a flow rate of 30 mL/min. The oven temperature was 60 °C for 5 min, from 60 to 190 °C at 5 °C/min and held for 5 min, and from 190 to 290 °C at 15 °C/min and held for 15 min. The injector and detector temperatures were 250 and 280 °C, respectively. The identification of the essential oil compounds was made by retention times and confirmed by the GC interfaced with an Agilent 5975C VL Mass Selective Detector with Triple-Axis Detector. The GC operating conditions were the same as above, but the sample injection (2 µL) was manual. The GC-MS interface temperature was 280 °C and an electron impact (EI) ionization of 70 eV was used. Full-scan masses from 50 to 600 *m*/*z* were recorded with the MassHunter Workstation Software v. B.07.05 (Agilent). The identification of compounds was made by comparing MS spectra obtained by analyzing pure reference compounds under the same operating conditions, and their identity was confirmed by matching the experimental MS spectra with reference spectra in the NIST14 library (Agilent).

### 2.4. Materials

Phospholipon 90G (≥94% soy phosphatidylcholine; P90G) was from Lipoid GmbH (Ludwigshafen, Germany). Polysorbate 80 (Tween 80) was from Galeno Srl (Carmignano, Prato, Italy). All other products, unless otherwise specified, were from Sigma-Aldrich/Merck (Milan, Italy).

### 2.5. Vesicle Preparation

Liposomes were prepared by dispersing P90G in ultrapure water, with or without the extract (essential oil or hydrolate) from pistachio stalks (Table 1). Transfersomes were prepared by adding Tween 80 to the dispersion of P90G in water, with or without the extract (essential oil or hydrolate) (Table 2). The dispersions were sonicated (10 cycles, 5 s ON/2 s OFF + 3 cycles, 3 s ON/2 s OFF) using a Soniprep 150 plus ultrasonic disintegrator (MSE Crowley, London, UK).

### 2.6. Vesicle Characterization

The average diameter, polydispersity index, and zeta potential of the liposomes and transfersomes were determined by dynamic and electrophoretic light scattering techniques using a Zetasizer nano-ZS (Malvern Panalytical, Worcestershire, UK). The vesicle dispersions were diluted with water prior to the analyses. The three parameters were monitored for 90 days to evaluate the storage stability (at 4 °C) of the nanoformulations.

The *P. vera* liposomes and transfersomes were purified by dialysis using 12–14 kDa Spectra/Por^®^ tubing (Spectrum Laboratories Inc., Breda, The Netherlands). Two mL of each sample was dialyzed against water (2 L) for 2 h to allow the removal of the non-incorporated extract components. Both non-dialyzed and dialyzed samples were diluted with methanol (1:100 *v*/*v*) and analyzed by LC-MS, as described in Section 2.2. The entrapment efficiency (EE) was calculated as the percentage of peak area of the main compounds (i.e., naringenin, myristic acid, catechin) detected in dialyzed vs. non-dialyzed samples.

### 2.7. Cell Culture and Treatments

Human monoblastic leukemia cell line U937 (Interlab Cell Line Collection of San Martino hospital (Genoa, Italy), HTL94002) were grown and differentiated to macrophages with phorbol 12-myristate 13-acetate (PMA; 10 ng/mL). Human embryonic kidney cell line HEK293 (Sigma-Aldrich, St. Louis, MO, USA) were maintained in Dulbecco’s Modified Eagle Medium (DMEM, Thermo Fisher Scientific) plus 10% fetal bovine serum (FBS; Corning, NY, USA), 2 mM *L*-glutamine, 100 U/mL penicillin, and 100 μg/mL streptomycin in a humidified 5% CO_2_ atmosphere at 37 °C.

U937/PMA and HEK293 cells were treated with 100 ng/mL or 500 ng/mL of *P. vera* essential oil dissolved in ethanol or the hydrolate dissolved in water, or formulated in the liposomes/transfersomes and diluted with the culture medium to reach the above concentrations of the extracts. U937/PMA cells, where appropriate, were stimulated with lipopolysaccharide from *Salmonella enterica* serotype typhimurium (LPS, Sigma-Aldrich, Milan, Italy; 200 ng/mL). The vesicle formulations were sterilized by using 0.22 µm syringe filters (STARLAB, Milan, Italy). During the study, all working cell culture stocks were periodically tested for mycoplasmal, bacterial, and fungal contamination.

### 2.8. Cell Viability Assay

U937 and HEK293 cells were seeded into 96-well plates (8 × 10^4^ and 1.5 × 10^4^ cells/well, respectively) and treated with the *P. vera* extracts in solutions or formulated in liposomes and transferosomes (100 ng/mL or 500 ng/mL). After 72 h, cell viability was estimated by a CellTiter-Glo^®^ 2.0 Assay (Promega, Madison, WI, USA). The latter determines the number of viable cells in culture by quantifying ATP, which indicates the presence of metabolically active cells. The CellTiter-Glo^®^ 2.0 reagent (100 μL) was added to the cells and mixed for 2 min on an orbital shaker to ensure cell lysis. Luminescence was measured using a microplate reader (GloMax, Promega, Madison, WI, USA) after 10 min of incubation at room temperature.

### 2.9. Western Blotting

Cell lysates were harvested and subjected to immunoblot analysis as previously described [24]. The method is based on SDS-PAGE followed by protein transfer to nitrocellulose membrane and treatment with specific antibodies. It is a widely used method with a remarkable reproducibility. The following specific antibodies against ATP citrate lyase (ab157098, Abcam, Cambridge, MA, USA) or anti-*β*-actin (ab8227, Abcam) were used. After 1-h incubation with Goat anti-Rabbit IgG-HRP secondary antibody (Santa Cruz Biotechnology, Santa Cruz, CA, USA), the immunoreaction was detected by using the WesternBright ECL horseradish peroxidase substrate (Advansta, Menlo Park, CA, USA) in a ChemiDoc XRS Detection System equipped with the Image Lab Software version 5.2.1 (Bio-Rad Laboratories, Hercules, CA, USA) for image acquisition and densitometric analysis. Representative western blots from three independent experiments with similar results are shown in the Figures.

### 2.10. ACLY Activity

U937/PMA cells were treated with LPS for 3 h in the presence or absence of *P. vera* extracts in solutions or in liposomes or in transfersomes (100 ng/mL). The cell lysate was prepared as previously described [25]. ACLY activity was measured via the malate dehydrogenase–coupled assay [26]. The cell lysate (150 μg) was added to a reaction mixture containing 50 mM Tris-HCl (pH 8.0), 10 mM magnesium chloride, 1.9 mM dithiothreitol, 0.15 mM NADH, 0.07 mM CoA, 1 mM ATP, 2 mM potassium citrate, and 3.3 units/mL malic dehydrogenase. The reaction was started by adding ATP, and the NADH oxidation was measured at 340 nm (25 °C). The ACLY activity was determined by normalization to the protein concentration and expressed as a percentage of the control.

### 2.11. ROS, NO• and PGE_2_ Detection

U937/PMA cells were stimulated with LPS in the presence or absence of *P. vera* extracts in solutions or in liposomes or in transfersomes (100 ng/mL) to measure ROS and NO• levels. Where indicated, the cells were treated also with 5 mM sodium malate (Sigma-Aldrich, Milan, Italy) or 500 μM NADPH (Sigma-Aldrich). After 24 h, ROS and NO• levels were measured by using 6-Carboxy-2′,7′-Dichlorodihydrofluorescein Diacetate (DCF-DA, Thermo Fisher Scientific, Milan, Italy) and 4-Amino-5-Methylamino-2′,7′-Difluorofluorescein Diacetate (DAF-FM Diacetate, Thermo Fisher Scientific), respectively.

For PGE_2_ quantification, the cells were exposed to *P. vera* extracts in solutions or in liposomes or in transfersomes (100 ng/mL) for 1 h and, where indicated, co-treated with 5 mM sodium acetate (Sigma-Aldrich); then, inflammation was induced by adding LPS. After 48 h, the PGE_2_ concentration was measured by using the DetectX^®^ Prostaglandin E_2_ Immunoassay Kit (Arbor Assays, AnnArbor, MI, USA).

### 2.12. Cellular Uptake of P. vera Extracts by LC–MS Analysis

Cellular uptake of *P. vera* extracts was determined by seeding U937 cells into 6-well plates (2.5 × 10^5^ cells/well) and treating them with the extracts in solutions or in liposomes or in transfersomes (100 ng/mL). After 24 h, the cells were pelleted at 1200 rpm for 5 min and supernatants were removed. A total of 500 µL of 40% MeOH with 0.1% *v*/*v* formic acid was added to the cell pellets, which were sonicated (1 min on/1 min off for 10 min), mixed, and placed on ice for 15 min to lyse the cells and extract their content. Lysates were centrifuged at 1500 rpm for 5 min and supernatants were collected for LC–MS analysis. Naringenin, myristic acid, and catechin cytoplasmic contents (as marker compounds reported as area of the chromatographic peaks of the protonated ions) were determined by LC-MS using the same instrument, column, and conditions used for the extract analysis to determine entrapment efficiency (Section 2.2), as per our previous work [27]. Data were acquired in positive full-scan mode.

### 2.13. Statistical Analysis

Results are reported as mean values ± standard deviations (SD) of, at least, three independent experiments run in triplicate. For pairwise comparisons, Student’s *t* test was performed. For more than two groups, one-way ANOVA followed by Dunnett’s multiple comparison tests was used. Differences were considered significant (*p* < 0.05), very significant (*p* < 0.01), and highly significant (*p* < 0.001). The statistical methods are detailed in Figure captions.

## 3. Results

### 3.1. Phytochemical Profile of P. vera Extracts

**Figure 1:**
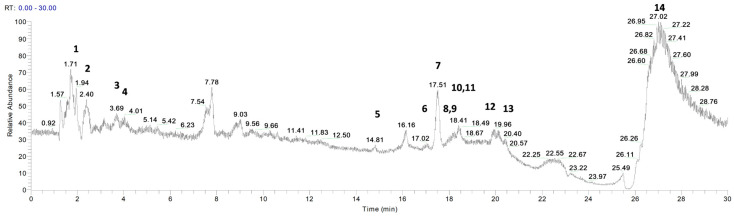
LC-ESI/Orbitrap/MS chromatogram of *P. vera* essential oil acquired in positive ion mode. Peaks identification is given in Table 3.

**Figure 2:**
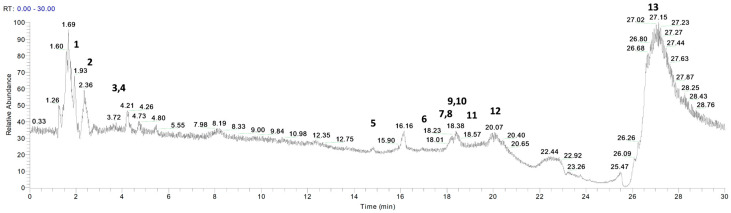
LC-ESI/Orbitrap/MS chromatogram of *P. vera* hydrolate acquired in positive ion mode. Peaks identification is given in Table 4.

*P. vera* extracts were analyzed by LC- and GC-MS to identify the relevant components. Figure 1 and Figure 2 show the chromatograms for the essential oil and the hydrolate, respectively. Table 3 and Table 4 list the metabolites identified with their retention times, chemical formulae, molecular weights, MS fragments, MS/MS results, and mass errors (∆ ppm).

Fourteen compounds were identified by comparing the *m*/*z* values in the total ion current (TIC) and the fragmentation patterns with those described in the literature [17,18,19,20,21,22,23].

In agreement with previous findings, the most abundant metabolites identified in the extracts were α-amino acids (tryptophan, tyrosine), hydroxycinnamic acids (vanillic acid, *p*-coumaric acid, sinapinic acid, caffeic acid, cinnamic acid and ferulic acid), saturated fatty acid (myristic acid), flavone (luteolin), flavan-3-ol (catechin), terpene (α-pinene), vitamin (ascorbic acid), and flavanone (naringenin) [22,23,28,29,30,31,32]. Of note, α-pinene was the only compound detected in the essential oil and not in the hydrolate (Figure 1 and Figure 2).

**Figure 3:**
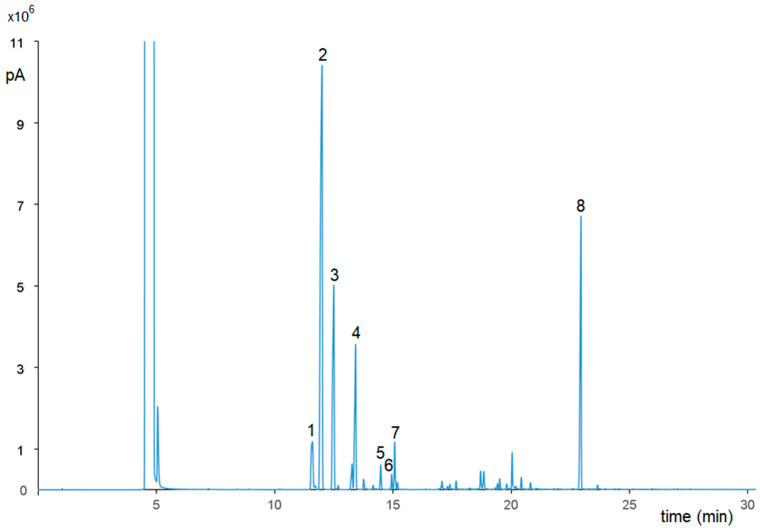
GC-FID chromatogram of *P. vera* essential oil. Peaks labelled in this figure are assigned from Table 5.

Figure 3 shows, by way of example, the chromatogram (GC-FID) of *P. vera* essential oil under the conditions described in Section 2.3. Eight compounds were identified (Table 5). The prevalent chemical components are monoterpenes, such as α-pinene (40.44%), camphene (15.89%), and bornyl acetate (15.53%). The distribution of all the compounds identified (in % area, obtained from the average of three replicates), inclusive of the non-priority ones, is shown in Table 5.

### 3.2. Vesicle Characterization

The liposomes and transfersomes were characterized for mean diameter, polydispersity, and zeta potential. To evaluate the extracts’ impact on the vesicles’ characteristics, the liposomes and transfersomes loaded with the *P. vera* extracts were compared, respectively, with the empty liposomes and empty transfersomes (Table 6).

The mean diameter of the empty liposomes was 85 nm and decreased upon incorporation of the essential oil (77 nm; *p* < 0.01), and the PI significantly improved, passing from 0.30 to 0.19, indicative of an excellent size homogeneity of these vesicles. Therefore, the essential oil induced the formation of smaller vesicles with a more uniform size distribution. The incorporation of the hydrolate, on the other hand, did not significantly alter the mean diameter (82 nm) nor the polydispersity (0.28; *p* > 0.05 vs. empty liposomes). The zeta potential of the empty liposomes was approximately −12 mV and remained unchanged upon incorporation of both extracts (Table 6).

The mean diameter of the empty transfersomes was significantly larger than that of the empty liposomes (95 vs. 85 nm), but the PI significantly improved, indicating a better homogeneity of the transfersomes (Table 6). These differences are due to the presence of the hydrophilic surfactant Tween 80. The incorporation of the two extracts in the transfersomes did not significantly alter the values of the three parameters analyzed, with two exceptions: the mean diameter of the transfersomes containing the hydrolate was smaller; and the PI of the transfersomes containing the essential oil was lower than those of the empty transfersomes. The zeta potential values for the transfersomes were slightly more negative than those of the corresponding liposomes (approximately −15 mV; Table 6).

The entrapment efficiency (EE) was calculated as a function of three abundant compounds of the extracts, naringenin, myristic acid, and catechin, detected in dialyzed vs. non-dialyzed *P. vera* liposomes and transfersomes. The values were well above 80% for the three compounds of the essential oil and the hydrolate, in both liposomes and transfersomes (Table 7).

The physical stability of the liposomes and transfersomes was evaluated by analyzing their mean diameter, polydispersity index, and zeta potential during storage. No signs of alterations were detected for transfersomes over 90 days (Table 8). The liposomes, on the other hand, were not stable for more than 30 days.

### 3.3. Cell Viability

**Figure 4:**
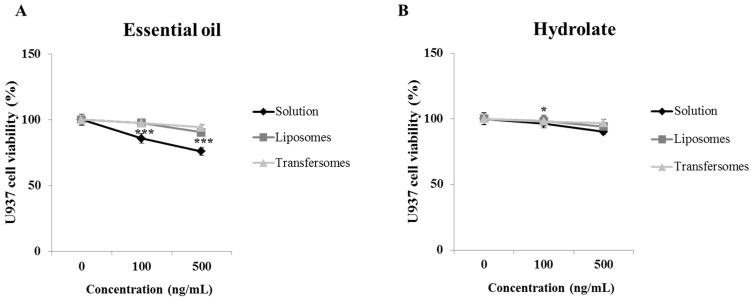
Effect of *P. vera* essential oil (**A**) and hydrolate (**B**) on the viability of U937 cells exposed to the extracts in solutions or in liposomes or transfersomes at the indicated concentrations (100 ng/mL or 500 ng/mL) for 72 h. Results are expressed as means ± SE of three separate experiments (one-way ANOVA followed by Dunnett’s multiple comparison test: *** *p* < 0.001, * *p* < 0.05 vs. untreated cells 0 ng/mL).

Firstly, U937 and HEK293 cell viability was assessed after treatment with the *P. vera* extracts in solutions or in liposomes or transfersomes. After 72 h, the essential oil caused a decrease in the U937 viability by 14 and 24% compared to untreated cells at concentrations 100 ng/mL or 500 ng/mL, respectively (Figure 4A). The hydrolate showed less toxicity than the essential oil, inducing a 10% reduction in viability at the higher concentration (500 ng/mL; Figure 4B). From the Figure, it is evident how the vesicle formulations abolished the toxicity of the essential oil almost completely and even more of the hydrolate. The extracts were more toxic to HEK293 cells. In particular, the essential oil reduced cell viability by 50% at the highest concentration (500 ng/mL; Figure S1A), while the hydrolate by 28% compared to untreated cells (Figure S1B). Again, only a slight effect was seen on cell viability when the nanoformulations were used, particularly liposomes. In light of these results, a 100 ng/mL concentration of each extract was chosen to evaluate the immune-modulating activity.

### 3.4. Effect on ATP Citrate Lyase Expression and Activity

The metabolic enzyme ACLY, which catalyzes the crucial reaction linking glucose catabolism and lipogenesis, is a key player during macrophages activation in response to different stimuli such as gram-negative bacterial endotoxin LPS, pro-inflammatory cytokines tumour necrosis factor α (TNFα) and interferon γ (IFNγ). Upon LPS-stimulation, ACLY is rapidly activated to induce pro-inflammatory genes reprogramming and, in turn, supports the production of inflammatory mediators [10].

**Figure 5:**
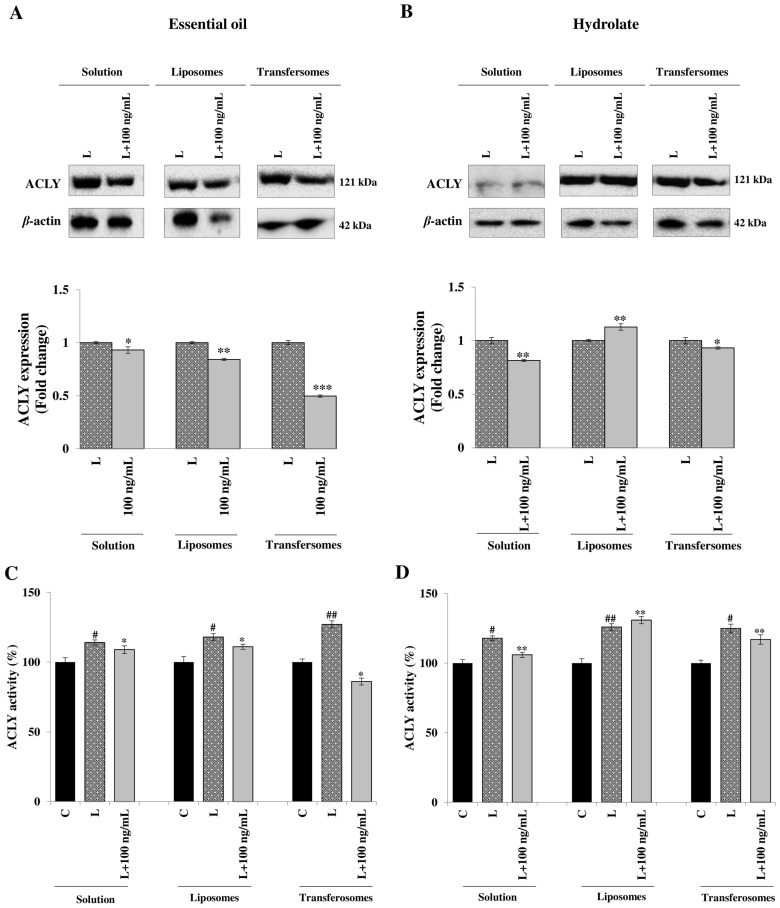
Effect of *P. vera* extracts on ACLY expression and activity. U937 cells were stimulated with LPS in the absence (L) or in the presence of *P. vera* essential oil or hydrolate in solutions, in liposomes, or in transfersomes. (**A**,**B**) Specific antibodies detected ACLY and β-actin; the protein expression was expressed as optical density ratio for ACLY versus β-actin. In bar charts, ACLY expression levels in LPS samples (L) were taken as 1, and the other values were expressed in proportion to the LPS. Student’s ***t*** test was used for pairwise comparisons (* *p* < 0.05, ** *p* < 0.01, *** *p* < 0.001). (**A**,**B**) Western blotting data are representative of at least three independent experiments with similar results. (**C**,**D**) ACLY activities are shown as means ± S.D. of three experiments. Unstimulated cells were used as control (**C**, black bars). Statistical significance of differences was evaluated by using one-way ANOVA followed by Dunnett’s multiple comparison test, comparing treatments with LPS-stimulated cells (* *p* < 0.05, ** *p* < 0.01). Differences between C (control) and L (LPS) were significant (^#^ *p* < 0.05, ^##^ *p* < 0.01).

The evaluation of the effect on ACLY expression was the starting point in the investigation of the immune-modulating potential of the extracts from *P. vera* stalks, free or formulated in liposomes or transfersomes. In LPS-stimulated U937 cells, the hydrolate was more effective than the essential oil in bringing down ACLY expression (Figure 5A,B). The formulation in liposomes improved the ability of the essential oil to inhibit ACLY, but the best result was obtained when it was formulated in the transfersomes, with a reduction of ACLY expression of about 50% (Figure 5A and Figure S2A). Conversely, the nanoformulation worsened the performance of the hydrolate, with liposomes giving an increase in the ACLY expression of about 15% (Figure 5B and Figure S2B).

Similar results were obtained when ACLY activity was measured. After LPS treatment, ACLY activity increased from 16 to 24% (Figure 5C,D). In the presence of the essential oil, either in solution or in liposomes, a small decrease in ACLY activity was shown, while a more evident decrease was apparent with transfersomes, reaching even lower levels than those of the control (unstimulated cells (Figure 5C)). On the contrary, the hydrolate was more effective in its free form than in the nanocarriers; the solution induced a 11% decrease in ACLY enzymatic activity (Figure 5D), whereas the liposomes did not decrease ACLY activity, and transfersomes induced a 7% decrease (Figure 5D).

### 3.5. Effect on ROS and NO• Production

**Figure 6:**
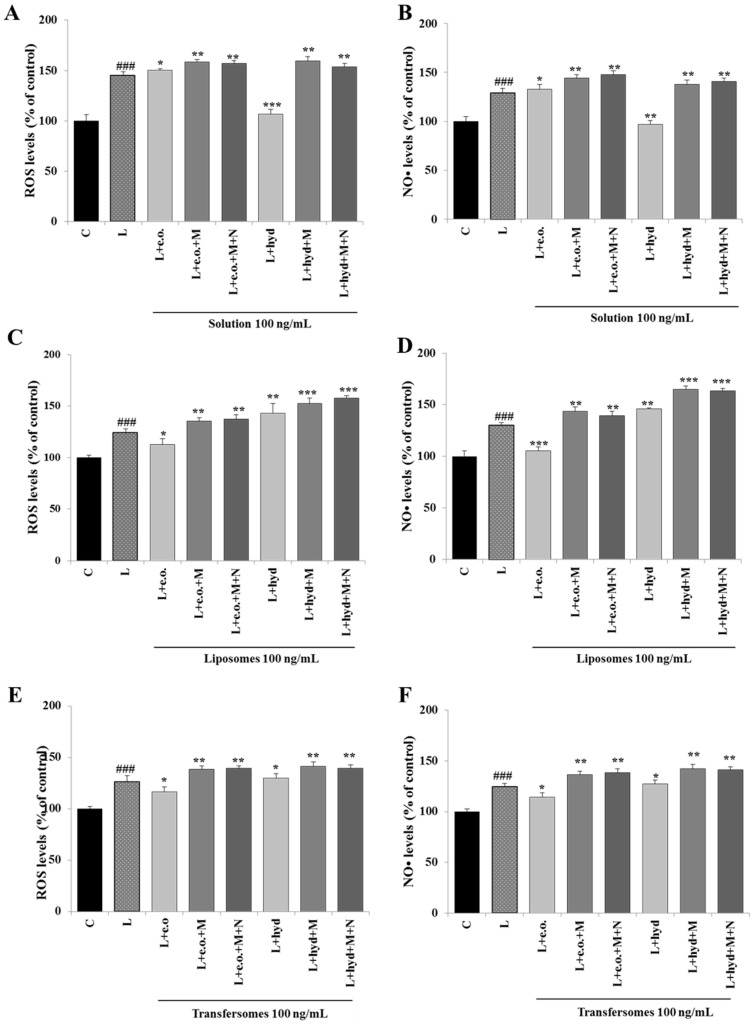
Effect of *P. vera* extracts on ROS and NO• production. U937 cells were stimulated with LPS in the absence (L) or in the presence of *P. vera* essential oil or hydrolate in solutions, or in liposomes or transfersomes, alone or with malate and NADPH. After 24 h, ROS (**A**,**C**,**E**) and NO• (**B**,**D**,**F**) levels were measured and expressed as the percentage of unstimulated control cells (C, 100%). Mean values ± SD of three independent experiments with at least five replicates each are presented. Statistical significance of differences was evaluated by using one-way ANOVA followed by Dunnett’s multiple comparison test, comparing treatments with LPS-stimulated cells (* *p* < 0.05, ** *p* < 0.01, *** *p* < 0.001). Differences between C and L were significant (^###^ *p* < 0.001). C: control; L: LPS; e.o.: *P. vera* essential oil; hyd: *P. vera* hydrolate; M: malate; N: NADPH.

Since *P. vera* extracts affected the expression and activity of ACLY, which supplies NADPH for the biosynthesis of ROS and NO•, we next focused on these inflammatory mediators. We observed enhanced releases of both ROS and NO• when U937 cells were stimulated with LPS (L, Figure 6). *P. vera* essential oil did not affect ROS (Figure 6A) nor NO• (Figure 6B), while the hydrolate reduced ROS and NO• to levels comparable with those of unstimulated cells (C; Figure 6A,B). When the essential oil was formulated in the nanocarriers, we detected a better inhibitory capacity toward ROS and NO• inflammatory mediators. A 10% reduction in ROS levels and 20% reduction in NO• levels were found for the liposomes (Figure 6C,D). Similar results were obtained with transfersomes, with a significant decrease in ROS and NO• levels by 10% compared to LPS-treated U937 cells (Figure 6E,F). In contrast, when the hydrolate was formulated in transfersomes, no effect was observed on ROS and NO• (Figure 6E,F), and with liposomes, an increase was seen (Figure 6C,D) compared to LPS-stimulated U937 cells.

The addition of exogenous malate and NADPH, two metabolites downstream of ACLY, led to a marked increase of both ROS and NO• by reverting ACLY inhibition. As shown in all the panels of Figure 6, the addition of malate, alone or combined with NADPH, induced an increase in ROS and NO• levels in LPS-stimulated U937 cells treated with *P. vera* extracts, both the essential oil and the hydrolate, in solutions or in liposomes or transfersomes. Therefore, the lowering of ROS and NO• levels induced by *P. vera* extracts is ACLY-mediated in LPS-treated U937 cells.

### 3.6. Effect on PGE_2_ Secretion

**Figure 7:**
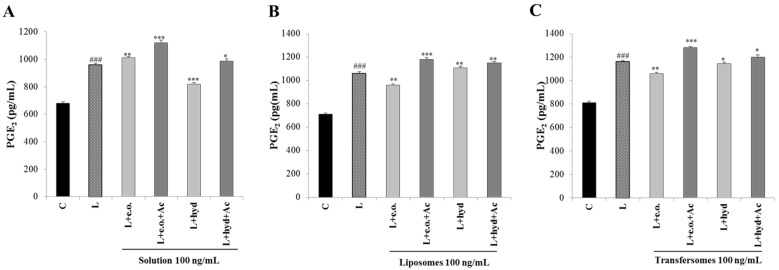
Effect of *P. vera* extracts on PGE_2_ production. U937 cells were stimulated with LPS in the absence (L) or in the presence of *P. vera* essential oil or hydrolate in solutions (**A**), or in liposomes (**B**) or transfersomes (**C**), alone or plus acetate. After 48 h, PGE_2_ levels were evaluated. Mean values ± SD of three independent experiments with at least three replicates each are presented. Statistical significance of differences was evaluated by using one-way ANOVA followed by Dunnett’s multiple comparison test, comparing treatments with LPS-stimulated cells (* *p* < 0.05, ** *p* < 0.01, *** *p* < 0.001). Differences between C and L were significant (^###^ *p* < 0.001). C: control; L: LPS; e.o.: *P. vera* essential oil; hyd: *P. vera* hydrolate; Ac: acetate.

Finally, we focused on PGE_2_, the other inflammatory mediator downstream of the citrate pathway. A reduction in PGE_2_ levels was observed following the treatment with the hydrolate solution (15% compared to LPS-stimulated cells; Figure 7A). The essential oil induced a reduction in PGE_2_ levels only when nanoformulated. In particular, we recorded a reduction of 10 and 9% compared to LPS-stimulated cells in liposomes and transfersomes, respectively (Figure 7B,C). The addition of acetate increased PGE_2_ levels (Figure 7A–C). The decrease in PGE_2_ production is most likely associated with a reduced availability of the precursors of PGE_2_ synthesis, such as acetate that can be converted to acetyl-coA by acetyl-coA synthase. In fact, the exogenous acetate abolished the ACLY-mediated inhibition of the PGE_2_ production.

### 3.7. Intracellular Accumulation of P. vera Extracts

**Figure 8:**
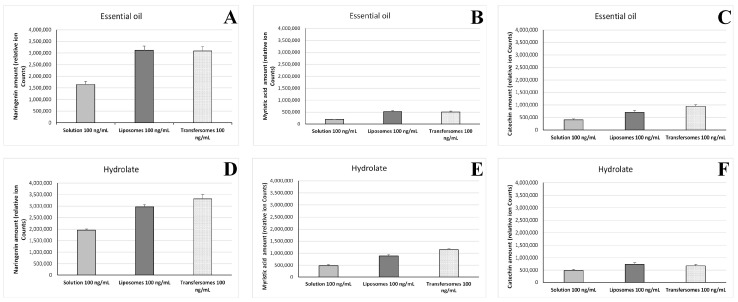
Amount of *P. vera* extracts, reported as areas of the chromatographic peaks of naringenin (**A**,**D**), myristic acid (**B**,**E**) and catechin (**C**,**F**), accumulated in U937 cells after exposure to the essential oil (100 ng/mL) or the hydrolate (100 ng/mL) in solutions, liposomes, and transfersomes. Results are reported as means of three replicates ± standard deviation.

LC-MS analysis was performed to quantify the intracellular accumulation of marker compounds (naringenin, myristic acid, catechin) of the *P. vera* essential oil and hydrolate after exposure of the cells to the vesicle formulations. Furthermore, a possible enhancement due to the nanoincorporation was studied by comparison with solutions of the extracts. The results indicate that the intracellular amount of the three compounds was approximately two-fold higher when the extracts were delivered by the liposomes and transfersomes (Figure 8).

## 4. Discussion

The incorporation of plant extracts in nanocarriers is a promising strategy to overcome the problems that often affect natural compounds, such as toxicity, instability, poor solubility, and low bioavailability. This, in turn, would improve their bioactivity. To the best of our knowledge, this is the first study that reports the nanoformulation and biological activities of two extracts obtained from the parts of pistachios that are usually discarded as waste. Twenty-one compounds with known biological relevance were identified in the essential oil and thirteen in the hydrolate. The compounds belong to structurally different metabolic classes: α-amino acids, hydroxycinnamic acids, saturated fatty acids, flavones, flavan-3-ols, terpenes, vitamins, and flavanones. The extracts were formulated in liposomes and transfersomes. The nanoformulations were produced by an easy, organic, solvent-free procedure involving the sonication of the phospholipid and the extracts dispersed in an aqueous medium. Both the liposomes and transfersomes were small in size, and capable of loading the two extracts efficiently, rewarding the choice of these nanocarriers. The mean diameter and the PI of the liposomes decreased following the incorporation of the essential oil, indicating an excellent dimensional homogeneity of these vesicles.

In another study, the average size of liposomes with a hydroalcoholic extract from *P. vera* was compared to that of the empty liposomes, and significant differences were observed. The liposomes loaded with the extract were significantly smaller and showed a higher homogeneity [33]. These results align with our findings, showing that the incorporation of pistachio extract can reduce the size of the vesicles and improve their characteristics.

The prepared transfersomes were larger and more homogeneous than liposomes. These differences are ascribed to the presence of the surfactant Tween 80. Moreover, transfersomes were found to be more stable than liposomes, as they showed no changes in size, homogeneity, and charge after 90 days from the preparation.

The choice of the vesicle formulations has been rewarded with an excellent entrapment efficiency for both the liposomes and transfersomes. One of the tree compounds quantified, naringenin, was best entrapped in the vesicles than the other two compounds, myristic acid and catechin.

Both the liposomes and the transfersomes were safe because of their almost null effect on cell viability at the tested concentrations. Importantly, they abolished the cytotoxicity of the essential oil. Both the essential oil and the hydrolate showed immune-modulating activity in LPS-stimulated U937/PMA cells. Notably, the incorporation in transfersomes was an effective approach to strongly increasing the activity of the essential oil on activated macrophages. The free hydrolate and the nanoformulated essential oil reduced LPS-induced ROS and NO• production. Interestingly, this effect was mediated by the immunometabolic citrate pathway, since treatments with NADPH and malate—metabolites downstream of ACLY [34]—reverted this phenotype. Moreover, the essential oil in transfersomes inhibited both ACLY gene expression and activity more than the free hydrolate. Further, the addition of acetate abolished the reduction of PGE_2_ secretion induced by the extracts. Therefore, our results highlight the role of *P. vera* extracts in modulating macrophage function through the immunometabolism. Indeed, the activation of the citrate pathway, of which ACLY is a part, is critical during macrophage activation in response to different stimuli, such as endotoxin LPS [35,36]. Thus, the *P. vera* extracts can affect the production of inflammatory mediators by acting on immunometabolism. Furthermore, the incorporation in transfersomes potentiated the immune modulation activity of *P. vera* essential oil.

## 5. Conclusions

The findings of this study show that the nanoformulations, especially transfersomes, developed for the *P. vera* extracts, especially the essential oil, hold promise for the modulation of the function of activated macrophages, which play a key role in responding to inflammation and initiating wound healing.

## Figures and Tables

**Table 1:** Composition of the liposome formulations.

	P90G	*P. vera* Extract	H_2_O
Empty liposomes	90 mg/mL	-	2 mL
*P. vera* liposomes	90 mg/mL	10 mg/mL	2 mL

**Table 2:** Composition of the transfersome formulations.

	P90G	*P. vera* Extract	Tween 80	H_2_O
Empty transfersomes	90 mg/mL	-	5 mg/mL	2 mL
*P. vera* transfersomes	90 mg/mL	10 mg/mL	5 mg/mL	2 mL

**Table 3:** Metabolites identified in *P. vera* essential oil via LC-ESI/Orbitrap/MS/MS (positive mode).

Peak No.	Compound	Rt (min)	Molecular Formula	Molecular Weight	[M+H]^+^	Δ Ppm	MS/MS
1	Vanillic acid	2.06	C_8_H_8_O_4_	168.18	169	0.18	142, 97
2	*p*-coumaric acid	2.52	C_9_H_8_O_3_	164.05	165	−0.31	147, 82
3	Sinapinic acid	3.60	C_11_H_12_O_5_	224.07	225	0.71	207
4	Myristic acid	3.73	C_14_H_28_O_2_	228.21	229	−0.21	225, 191
5	Caffeic acid	14.57	C_9_H_8_O_4_	180.04	181	0.41	181, 163
6	Catechin	16.95	C_15_H_14_O_6_	290.08	291	0.38	245, 151, 109
7	α-pinene	17.49	C_10_H_16_	136.24	137	0.24	109, 95, 81
8	Ascorbic acid	17.96	C_6_H_8_O_6_	176.04	177	−0.43	177, 159
9	Tryptophan	17.99	C_11_H_12_N_2_O_2_	204.09	205	0.39	205, 144
10	Naringenin	18.36	C_15_H_12_O_5_	272.07	273	0.57	255, 244,95
11	Cinnamic acid	18.41	C_9_H_8_O_2_	148.05	149	−0.34	131, 121, 65
12	Luteolin	18.83	C_15_H_10_O_6_	286.24	287	0.24	269, 243, 135
13	Ferulic acid	20.33	C_10_H_10_O_4_	194.06	195	−0.26	177
14	Tyrosine	26.88	C_9_H_11_NO_3_	181.07	182	0.36	182, 165, 136

**Table 4:** Metabolites identified in *P. vera* hydrolate via LC-ESI/Orbitrap/MS/MS (positive mode).

Peak No.	Compound	Rt (min)	Molecular Formula	Molecular Weight	[M+H]^+^	Δ Ppm	MS/MS
1	Vanillic acid	1.99	C_8_H_8_O_4_	168.18	169	0.17	142, 97
2	*p*-coumaric acid	2.50	C_9_H_8_O_3_	164.05	165	0.52	147, 82
3	Sinapinic acid	3.61	C_11_H_12_O_5_	224.07	225	0.73	207
4	Myristic acid	3.72	C_14_H_28_O_2_	228.21	229	−0.22	225, 191
5	Caffeic acid	15.56	C_9_H_8_O_4_	180.04	181	0.46	181, 163
6	Catechin	16.96	C_15_H_14_O_6_	290.08	291	0.22	245, 151, 109
7	Ascorbic acid	17.80	C_6_H_8_O_6_	176.04	177	−0.46	177, 159
8	Tryptophan	17.99	C_11_H_12_N_2_O_2_	204.09	205	0.29	205, 144
9	Naringenin	18.39	C_15_H_12_O5	272.07	273	0.61	255, 244
10	Cinnamic acid	18.47	C_9_H_8_O_2_	148.05	149	−0.57	131, 121, 65
11	Luteolin	18.22	C_15_H_10_O_6_	286.24	287	0.26	269, 135
12	Ferulic acid	20.33	C_10_H_10_O_4_	194.06	195	−0.62	177
13	Tyrosine	26.63	C_9_H_11_NO_3_	181.07	182	0.23	182, 165, 136

**Table 5:** Chemical components of *P. vera* essential oil. The values represent the means of three measurements. In all cases, relative standard deviations were <5%.

Peak No.	Compound	% Area
1	tricyclene	4.68
2	α-pinene	40.44
3	camphene	15.89
4	β-pinene	9.52
5	3-carene	1.23
6	*p*-cymene	0.25
7	limonene	2.24
8	bornyl acetate	15.53

**Table 6:** Characteristics of liposomes and transfersomes: mean diameter (MD), polydispersity index (PI), and zeta potential (ZP). Values represent the means ± SD (*n* > 10). *, **: values statistically different (*p* < 0.05 and *p* < 0.01, respectively) from empty liposomes; ^##^: values statistically different (*p* < 0.01) from *P. vera* essential oil liposomes; ^••^: values statistically different (*p* < 0.01) from *P. vera* hydrolate liposomes; °, °°: values statistically different (*p* < 0.05 and *p* < 0.01, respectively) from empty transfersomes.

	MD (nm ± SD)	PI ± SD	ZP (mV ± SD)
Empty liposomes	85 ± 4.3	0.30 ± 0.03	−12 ± 2.8
*P. vera* essential oil liposomes	** 76 ± 6.5	** 0.19 ± 0.03	−11 ± 2.6
*P. vera* hydrolate liposomes	82 ± 6.9	0.28 ± 0.01	−11 ± 1.8
Empty transfersomes	** 95 ± 6.1	** 0.24 ± 0.03	* −15 ± 2.1
*P. vera* essential oil transfersomes	^##^ 90 ± 9.6	° 0.20 ± 0.04	−13 ± 3.4
*P. vera* hydrolate transfersomes	°° 85 ± 5.1	^••^ 0.23 ± 0.01	^••^ −15 ± 2.8

**Table 7:** Entrapment efficiency (EE) based on peak areas of the main compounds identified in *P. vera* extracts.

Compound.	Formulation	EE % ± SD
Naringenin	*P. vera* essential oil liposomes	93 + 0.9
	*P. vera* hydrolate liposomes	94 + 1.5
	*P. vera* essential oil transfersomes	89 + 3.1
	*P. vera* hydrolate transfersomes	92 + 2.7
Myristic acid	*P. vera* essential oil liposomes	85 + 4.3
	*P. vera* hydrolate liposomes	84 + 1.9
	*P. vera* essential oil transfersomes	85 + 4.8
	*P. vera* hydrolate transfersomes	83 + 2.0
Catechin	*P. vera* essential oil liposomes	86 + 3.9
	*P. vera* hydrolate liposomes	83 + 1.4
	*P. vera* essential oil transfersomes	82 + 2.9
	*P. vera* hydrolate transfersomes	84 + 3.0

**Table 8:** Stability of *P. vera* transfersomes. Mean diameter (MD), polydispersity index (PI), and zeta potential (ZP) are reported. Values represent the means ± SD (*n* = 4).

	Time	MD (nm ± SD)	PI ± SD	ZP (mV ± SD)
Empty transfersomes	30 days	102 ± 3.0	0.26 ± 0.03	−14 ± 2.1
*P. vera* essential oil transfersomes		101 ± 7.9	0.18 ± 0.01	−13 ± 1.8
*P. vera* hydrolate transfersomes		89 ± 8.1	0.25 ± 0.01	−14 ± 1.1
Empty transfersomes	60 days	101 ± 0.5	0.21 ± 0.03	−11 ± 1.3
*P. vera* essential oil transfersomes		101 ± 5.0	0.17 ± 0.03	−14 ± 1.8
*P. vera* hydrolate transfersomes		91 ± 4.4	0.25 ± 0.05	−10 ± 5.0
Empty transfersomes	90 days	101 ± 4.9	0.22 ± 0.02	−9 ± 2.6
*P. vera* essential oil transfersomes		100 ± 7.9	0.18 ± 0.01	−13 ± 1.6
*P. vera* hydrolate transfersomes		96 ± 8.1	0.26 ± 0.02	−11 ± 4.1

## Data Availability

The data presented in this study are available within this article.

## Supplementary Materials

The following supporting information can be downloaded at: https://www.mdpi.com/article/10.3390/pharmaceutics15051540/s1, Figure S1: HEK293 viability (* *p* < 0.05, ** *p* < 0.01, *** *p* < 0.001). Figure S2: Full picture of ACLY western blotting for *P. vera* essential oil (A) and hydrolate (B).
