# Supplementary material for: Immunometabolism Modulation by Extracts from Pistachio Stalks Formulated in Phospholipid Vesicles
Source: Pharmaceutics. 2023 May 19;15(5):1540. doi: 10.3390/pharmaceutics15051540 (PMC10223882; doi:10.3390/pharmaceutics15051540)
Supplement: Supplementary file 1 [file pharmaceutics-15-01540-s001.zip › pharmaceutics-2367335-supplementary.pdf]

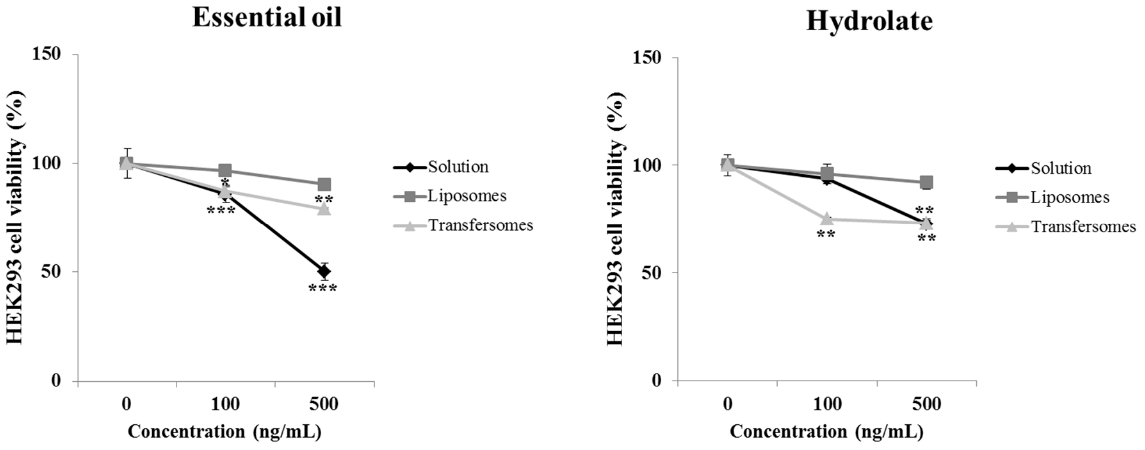

Figure S1. HEK293 viability (* $p < 0.05$, ** $p < 0.01$, *** $p < 0.001$).

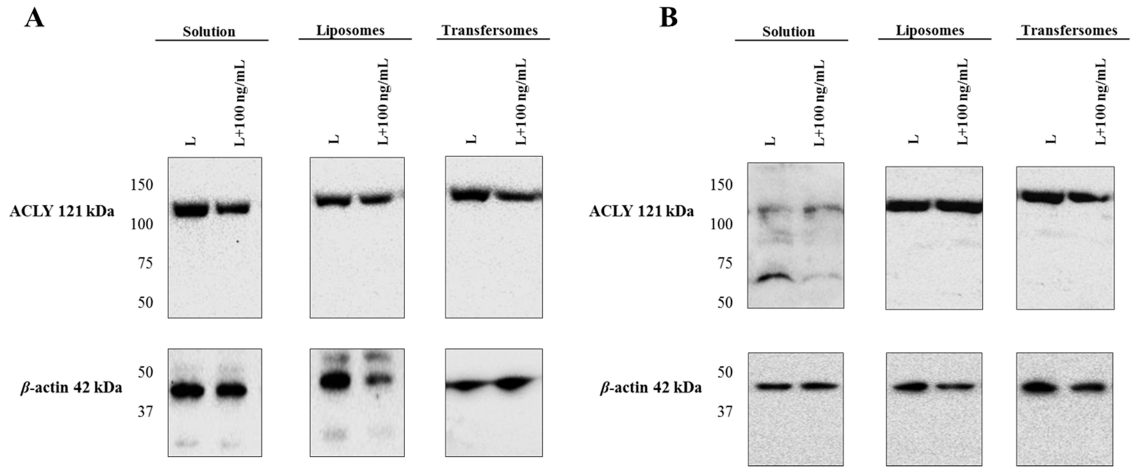

Figure S2. Full picture of ACLY western blotting for *P. vera* essential oil (A) and hydrolate (B).
